# Meningitis

**Published:** 1955-09

**Authors:** A. M. G. Campbell

**Affiliations:** Consultant Physician, United Bristol Hospitals


### MENINGITIS

BY

A. M. G. CAMPBELL, M.D., F.R.C.P.
*Consultant Physician, United Bristol Hospitals*

### INTRODUCTION

Inflammation of the meninges and brain in the past was always considered such a serious and fatal disease that its early diagnosis was not stressed sufficiently. Since the introduction of antibiotics it has been shown that mortality figures are in direct proportion to the delay in diagnosis and treatment. Therefore, the early diagnosis of meningitis and establishment of the organism, which has infected the brain, are vital.

Infection of the meninges may be *via* the blood stream, as is predominantly the case in meningococcal meningitis, or by a direct spread from neighbouring structures, such as infected bone or sinuses. Meningitis may also be due to chemical irritation from spinal anaesthetics or from virus and spirochaetal infection.

The onset of a meningitis can be extremely acute and is accompanied by generalized severe headache but, in particular, the headache is very often localized to the back of the neck. All the signs of a meningeal irritation depend on a reflex muscle spasm arising from stretching inflamed spinal roots, either in the cervical region accounting for neck rigidity or in the lumbar region accounting for Kernig's sign, a sign which does not allow of extending the flexed knee with hip flexion. It must be emphasized that any irritation of the meninges such as that caused by blood in a sub-arachnoid haemorrhage or by increased intracranial pressure associated with a cerebral tumour, may produce these signs. Again in certain febrile illnesses, particularly pneumonia and typhoid, and occasionally tonsillitis, these signs may be positive and account for the so-called case of meningism, which is thought to be due to a fall in the osmotic pressure of the serum and increased production of cerebrospinal fluid, but is not accompanied by cellular increase in the fluid.

The diagnosis of meningitis from other irritative lesions of the meninges can only be made by lumbar puncture, which is absolutely essential in any doubtful case of this kind, not only for diagnosis but to establish the organism and its sensitivity.

The early symptoms of meningitis differ slightly in children and adults. Dr. Corner has enlarged very fully on the difficulty of diagnosis in children and emphasized that headache is not such a predominant symptom in childhood as it is in adults. A certain number of adult cases will arrive in coma so that other causes of coma are a differential diagnosis in this disease. Other cases present with epilepsy or with some other odd symptoms which I will mention later in connection with tuberculous meningitis; one pneumococcal meningitis presented with sciatic pain.

All damage to the brain whether it is infective or traumatic leads to intellectual impairment during the course of the illness. In many people this intellectual impairment strikes at the very base of their personality and, even after recovery from the primary condition, they may become unduly worried about the question of brain damage and often a secondary anxiety state is grafted on to any organic disease which remains. If the individual previously had a somewhat neurotic personality, he is best treated by careful reassurance and graduated convalescence. It is particularly important to reassure him about his future attainments. These symptoms differ in no way from those shown by anxious people after a head injury.

Other possible complications of any meningitis are adhesions in the arachnoid space, which may produce constriction of the spinal cord, paralysis, and hydrocephalus.

Lastly, epilepsy can follow meningitis just as it can follow a head injury and it can come on several years after the acute attack; it is usually related to the severity of the initial illness and whether treatment has been instituted early.

### MENINGOCOCCAL MENINGITIS

Meningococcal meningitis is largely a disease of children, but it does attack young adults and in particular those living in the crowded conditions which occurs in wars. In the spring months, it can take an epidemic form. This was seen in 1915 and again in 1940. In the R.A.F. in 1940 I was in the position of looking after two wards of meningococcal meningitis. In this series in all we had 54 cases and 2 deaths. One patient died as the result of a fulminating attack accompanied by septicaemia, purpura and haemorrhage into the suprarenals within twenty-four hours of the symptoms developing. In this R.A.F. outbreak, we had two patients with deafness, which is due to the disease infecting the labyrinth. We had one case of herpes zoster supervening on the disease and two cases of meningococcal septicaemia associated with meningitis and producing arthritis.

### PNEUMOCOCCAL MENINGITIS

Pneumococcal meningitis is a disease of all ages, but occurs very frequently in adults. Its cause is usually an infection from the mastoid air cells, sinuses or middle ear, but quite a few cases are the result of trauma and a fracture of the inner wall of a sinus allowing infection to spread to the meninges. Another important cause is pneumococcal infection of the chest or the spread of infection from bronchiectasis or lung abscess. In the last group the occurrence of serious disease both in the chest and in the head undoubtedly account for many difficulties in treatment for many of the cases die as a result of their chest condition rather than from their meningitis, or a combination of both conditions. Sometimes the disease follows operation and two cases recently have followed operation for polypi in the antrum.

Before the days ot antibiotics, pneumococcal meningitis was almost always fatal, but, with the introduction of sulphonamides, the mortality was reduced to approximately 70 per cent, and with the addition of penicillin to the treatment, the outlook has been greatly improved and the mortality has been reduced to between 30 and 40 per cent. Results in certain Units, such as Oxford, have been strikingly good and the mortality has been as low as 24 per cent. The difficulty in comparing mortality figures in this disease is the association of other diseases, which is quite common with pneumococcal meningitis in elderly individuals.

The occurrence of this type of meningitis in the old-age group is quite striking and within the last year, I have treated two cases of this disease in patients over sixty-five years of age. The first recovered and the second died. The second case was sent to hospital late because the doctor imagined that the epileptic fit which the patient presented with, was due to cerebral vascular disease and not due to meningitis.

The rapidity of the onset of this type of meningitis is quite remarkable. For example, the wife of a doctor went to bed with ear-ache one night. She complained of ear-ache and headache the following morning and by 1.0 p.m. was found unconscious, with severe meningeal symptoms. Treatment with intrathecal penicillin was instituted at 4.0 p.m. and she made an uneventful recovery.

The second personal case was that of a boy of sixteen years of age, who was knocked on the head in the frontal region whilst playing football at 3.0 p.m. At 6.0 p.m. he complained of headache and was admitted to the Royal Infirmary, where a lumbar puncture was performed at 8.30 p.m. He was already semi-comatose and was found to have a purulent pneumococcal meningitis six hours after the blow. He also made an uneventful recovery. Unless treated promptly these fulminating cases will succumb very rapidly, so that early diagnosis is absolutely essential.

The question of therapy for pneumococcal meningitis does not intimately concern general practitioners. It suffices to say that sulphonamides and penicillin are both necessary; and penicillin should be given systemically and intra-thecally. Whilst meningococcal meningitis does extremely well on sulphonamides alone, penicillin is the sheet-anchor of the treatment of pneumococcal infections. The newer antibiotics are quite unnecessary unless the organism is resistant to penicillin, and it has actually been shown the aureomycin given with penicillin produces a worse recovery rate than penicillin alone.

Where the meningitis results from trauma due to infection entering through a dural tear, a fascial repair by a neurosurgeon is necessary to prevent relapses.

Sometimes the cause of infection remains unknown even after extensive investigations, and one such case in Bristol had six relapses in seven years, all responding to penicillin. This patient can now diagnose his meningitis within an hour of its onset. Another patient had a dural repair on one side and then relapsed and was found to have a tear on the other side.

Any therapy must be maintained until the cerebrospinal fluid regains near normality, and a lumbar puncture must be done after therapy has ceased to see that there is no recurrence. Sometimes loculi of infection occur in the ventricles and account for persisting unresponsive infections.

Rarely an abscess may develop in the brain and I would stress the fact that meningitis can accompany a localized abscess. If a meningitis fails to respond to adequate therapy or localizing signs of brain damage develop, particularly with a raised protein, an abscess should always be suspected. Air pictures, arteriography and electro-encephalograms should then be done.

It will be seen that any case of meningitis needs the most careful observation and contı ol. The position in these cases is always changing from day to day and any development of new symptoms will have to be met by new measures to avoid complications or to deal with these complications. Treatment, therefore, has become a highly specialized technical procedure demanding the co-operation of the bacteriological laboratory and the clinician. It c nnot, and should not, be carried out at home. In any Unit treating meningitis, the results of treatment of any form are very much rosier than they were ten years ago.

It remains to say one word about pyogenic meningitis arising from lumbar puncture. It is unfortunately true that pyogenic meningitis sometimes arises as a result of lumbar puncture or the introduction of a spinal anaesthetic. The sterility of lumbar puncture needles and lumbar puncture set is absolutely essential to avoid these serious accidents. If a patient persistently runs a temperature after a lumbar puncture or the neck is unduly stiff, the possibility of the development of a meningitis must always be considered.

### PREDOMINANTLY LYMPHOCYTIC MENINGITIS

Meningitis in which the cells in the cerebro-spinal fluid are predominantly lymphocytes can be classified as follows:

*Due to organism:*
1. Tuberculous meningitis
2. Syphilis
3. Leptospirosis

*Due to virus:*
1. Poliomyelitis
2. Mumps
3. Choriomeningitis
4. Glandular fever
5. Herpes simplex and zoster
6. Unknown cause

### TUBERCULOUS MENINGITIS

Dr. Beryl Corner has dealt with the disease in children, and it only remains to say that in adults the basic symptomatology and treatment are the same. The diagnosis can be difficult for the onset is very often insidious and it is not always realized that the disease can occur at any age. Examination of the fundi for choroidal tubercles is more important and easier in an adult than a child. Chest X-rays in adults often show that the disease is part of a miliary process.

One patient presented at the Bristol Eye Hospital with an ophthalmic herpes, which was a symptomatic manifestation of a severe tuberculous meningitis at the age of sixty-three.

On examination of the cerebrospinal fluid, the finding of the organism, and persistently low sugar rates are characteristic of the disease; but a low sugar in the cerebrospinal fluid is highly suggestive and treatment should be instituted with streptomycin, isoniazid and, if necessary, P.A.S. At this point, I would like to add that I am quite certain that intrathecal treatment should be persisted with unless there are very good contra-indications. The cases that have not been treated with intrathecal streptomycin on the assumption that the introduction of isoniazid has made this unnecessary have not been followed up sufficiently long to assess whether the relapse rate is as satisfactory as it is in those cases treated intrathecally.

We have had two interesting cases of pregnant women with tuberculous meningitis in the Bristol series. The first was treated in 1948–49; unfortunately she relapsed after delivery with acute pulmonary spread of infection and died. Her child lived and is perfectly fit and well at the moment, and I might quote a letter from Dr. Hartley received recently.

"The baby was transferred from Southmead Hospital to the Babies Home and was under my care during the first two years. She made uninterrupted progress being physically of very good standard and hearing and mentality in every way normal. Skin tests for T.B. during this period also remained negative.

"After two years she left the home, but I have kept in touch with her and have seen her on many occasions, and she is still very well and now at school."

This case is important as shown by the fact that the child did not suffer as a result of her mother's disease or from the treatment given during pregnancy, and the labyrinth of this child is quite normal.

We have recently treated a patient in the Bristol Royal Infirmary who was satisfactorily delivered by Professor Lennon (by a Caesarean section) of a baby weighing 8 lb. Both mother and child are now perfectly well.

Occasionally tuberculous meningitis is accompanied by endarteritis of the brain and cerebral thrombosis is apt to occur with hemiplegia, which may mask the tuberculous process.

You may ask if treatment entirely eradicates this disease. In the best hands there is recovery of 50 per cent of cases of tuberculous meningitis, but a certain number of these may relapse and unfortunately there is always a slight risk of a relapse in a case of tuberculous meningitis. On the other hand, Dr. Macrae at Ham Green Hospital treated a man in the fifties, who died eighteen months later of heart disease and a post mortem carried out at Exeter showed no evidence of tuberculosis of the brain.

We can now offer these sufferers a very reasonable hope of a satisfactory life and if care is used there is certainly no danger from the treatment itself. I have included tuberculous meningitis in the lymphocytic group although many cases show a polymorph response in the fluid, particularly in the early stages.

### SYPHILIS

This disease presenting as an acute meningitis and as part of a meningo-vascular syphilis is rare. The onset is usually more chronic and does not cause confusion with an acute meningitis, but occasionally syphilis can present in this way, resembling any other acute meningitic process.

In the last seven years, I have seen one such case. The disease was suggested by focal signs, ocular motor palsy and Argyll Robertson pupils, and confirmed by the Wassermann reaction, which was positive in blood and fluid.

### LEPTOSPIROSIS

Leptospirosis occurs round Bristol in the form of Weil's disease and Canicola fever. Weil's disease is usually contracted as a result of bathing in stagnant pools, which are frequented by rats, and in any meningitis in which there is a history of recent bathing, this infection should be suspected. The diagnosis is also suspected by suffusion of the conjunctivae and the development of jaundice. Canicola fever, which is contracted from dogs, is not such a severe disease and predominantly shows a lymphocytic meningitis. Contact with a sick dog, or for example, in one case, bathing in a pool in which there was a dead dog, may be responsible for infection. About fifteen cases occur a year in Bristol and both types can be diagnosed by agglutination reactions.

### ASEPTIC MENINGITIS

Meningitis in which no bacterium is to blame may be called "Aseptic". Wallgren has classified the criteria for aseptic meningitis as follows:

1. Acute meningeal onset. 2. Predominantly lymphocytic meningitis. 3. Absence of bacteria in the cerebrospinal fluid. 4. The usually short, more benign type of illness. 5. The absence of evidence of sinus, aural, septicaemic or traumatic origin.

### POLIOMYELITIS

By far the most important meningitis in this group is, of course, poliomyelitis and where paralysis does not occur the diagnosis is not always easy. One should always suspect the possibility of poliomyelitis in every case of lymphocytic meningitis, particularly when there is a history of an alimentary disturbance or a sore throat with the typical root pains. Lumbar puncture will not always help in the diagnosis. In a proven case of paralytic poliomyelitis it is unnecessary and possibly harmful; but in a doubtful case it is justifiable to exclude the possibility of bacterial meningitis.

The diagnosis of poliomyelitis will, of course, be thought about in a summer epidemic period and in particular in the months of July to October. But a thorough examination of the patient and a careful history is always necessary; in three cases that have previously been diagnosed as poliomyelitis, I have been able to reassure the relatives that the disease was merely mumps. In the first case, a recent contact with mumps proved helpful and in the other cases, slight enlargement of the parotid glands and testicular pain showed that the correct diagnosis was mumps and not poliomyelitis.

### HERPES SIMPLEX AND GLANDULAR FEVER

Both herpes simplex and glandular fever may be responsible for lymphocytic meningitis. In glandular fever, the Paul Bunnell test and blood count may be of help. Herpes can only be diagnosed as a result of agglutination reactions.

In a survey of lymphocytic meningitis occurring in the American Forces over a period of ten years, it was shown that 10 per cent of these cases could be accounted for by mumps; 10 per cent by leptospirosis; 5 per cent by herpes; 10 per cent were probably lymphocytic choriomeningitis, (a virus disease which many think is contracted from mice); the remaining 65 per cent could not be diagnosed on pathological grounds and were probably non-paralytic poliomyelitis. All that can be said is that if a lymphocytic meningitis occurs in the winter, it is less likely to be due to poliomyelitis than if it occurs in the summer. A careful history and a careful clinical examination will help in the diagnosis of most cases, while a further doubtful case may be diagnosed by referring blood and cerebrospinal fluid to the Central Virus Research Laboratories under the direction of Dr. McCallum of Colindale. Unless the diagnosis of poliomyelitis is established, every effort should be made to find the cause of the meningitis by pathological tests, as I have indicated, and by examination of the cerebrospinal fluid.

In conclusion, let me stress again that the most important point in meningitis is the need for early diagnosis and early treatment. The antibiotics, which we have at our disposal, will be wasted unless the diagnosis is made early and the treatment is instituted early and for sufficiently long in each case to control the disease. Every practitioner should be on the lookout for the very suggestive signs of neck rigidity or root irritation, and when in doubt the case should be referred to a hospital for specialized opinion. The recovery rate in most cases of meningitis, whatever their cause, is so good that the whole outlook of this once very serious condition has been changed in a decade.

